# The Phenotype of the *C9ORF72* Expansion Carriers According to Revised Criteria for bvFTD

**DOI:** 10.1371/journal.pone.0131817

**Published:** 2015-07-06

**Authors:** Eino Solje, Heidi Aaltokallio, Heli Koivumaa-Honkanen, Noora M. Suhonen, Virpi Moilanen, Anna Kiviharju, Bryan Traynor, Pentti J. Tienari, Päivi Hartikainen, Anne M. Remes

**Affiliations:** 1 Institute of Clinical Medicine–Neurology, University of Eastern Finland, Kuopio, Finland; 2 Institute of Clinical Medicine–Psychiatry, University of Eastern Finland, Kuopio, Finland; 3 Department of Psychiatry, Kuopio University Hospital, Kuopio, Finland; 4 Department of Psychiatry, South-Savonia Hospital District, Mikkeli, Finland; 5 Department of Psychiatry, North Karelia Central Hospital, Joensuu, Finland; 6 Department of Psychiatry, SOSTERI, Savonlinna, Finland; 7 Department of Psychiatry, SOTE, Iisalmi, Finland; 8 Department of Psychiatry, Lapland Hospital District, Rovaniemi, Finland; 9 Department of Neurology, Oulu University Hospital, Oulu, Finland; 10 Molecular Neurology, Research Programs Unit, University of Helsinki, Department of Neurology, Helsinki University Central Hospital, Helsinki, Finland; 11 Neuromuscular Diseases Research Unit, Laboratory of Neurogenetics, National Institute on Aging, National Institutes of Health, Bethesda, Maryland, United States of America; Brain Sciences Institute, Johns Hopkins University, Baltimore, Maryland, United States of America; 12 Department of Neurology, Kuopio University Hospital, Kuopio, Finland; Pasteur Institute of Lille, FRANCE

## Abstract

**Background:**

The *C9ORF72* expansion is one of the most common genetic etiologies observed with behavioural variant frontotemporal dementia (bvFTD). Revised diagnostic criteria for bvFTD (FTDC) were recently introduced but only a few studies have evaluated the accuracy of these criteria.

**Objective:**

The objective of the study was to evaluate the applicability of the FTDC criteria and assess the psychiatric history of these patients.

**Methods:**

The study examined 36 patients carrying the *C9ORF72* expansion and suffering from bvFTD (N = 32) or from bvFTD with motor neuron disease (bvFTD-MND, N = 4). Neuropsychological, neuropsychiatric, structural brain imaging and PET/SPECT data were evaluated.

**Results:**

We found 0.75 sensitivity (SD 0.44, 95%CI 0.57–0.87) for possible bvFTD and 0.64 (SD 0.44, 95%CI 0.57–0.87) for probable bvFTD. The sensitivity was even higher in bvFTD patients without MND, i.e., 0.81 for possible bvFTD and 0.69 for probable bvFTD. PET/SPECT was normal in 17.6% of scanned patients with bvFTD. A history of psychiatric symptoms (psychotic and/or mood symptoms) was detected in 61% of cases.

**Conclusions:**

The FTDC possible and probable bvFTD criteria seem to identify the majority of the *C9ORF72* expansion carriers with bvFTD, even though they exhibit only a limited number of behavioral criteria but a significant amount of psychiatric symptoms. The presence of a normal PET/SPECT does not exclude the possibility the *C9ORF72* associated bvFTD.

## Introduction

Frontotemporal lobar degeneration (FTLD) is a clinically, genetically and neuropathologically heterogeneous group of progressive syndromes especially affecting the frontal and temporal brain regions. FTLD can be separated into three major clinical subgroups. 1) The behavioural variant frontotemporal dementia (bvFTD) is characterized by personality changes, social dysfunction and executive deficit; the other two forms are language variants; 2) semantic variant primary progressive aphasia (svPPA) and 3) nonfluent variant primary progressive aphasia (nfvPPA). About 15% of patients with FTLD also develop concomitant motor neuron disease (MND) [1]. On the other hand, approximately 15% of the patients with amyotrophic lateral sclerosis develop FTLD [2].

The hexanucleotide repeat expansion in chromosome 9 open reading frame 72 (*C9ORF72)* has been claimed to be present in the majority of familial FTLD and MND cases [3,4]. In Finland, the frequency of the *C9ORF72* expansion associated FTLD is among the highest in the world and is present in nearly 50% of familial Finnish FTLD cases [4,5].

Rascovsky and colleagues noted that Neary’s 1998 consensus criteria for bvFTD [6] were somewhat out-of-date [7]. Thus, revised criteria for bvFTD were introduced in 2011 by the International Behavioural Variant FTD Criteria Consortium (FTDC), to meet these criticisms [8]. However, the sensitivity of these new criteria has been evaluated in only a few studies. In two neuropathological confirmed studies (total number of patients N = 293), the sensitivity for detecting possible bvFTD varied between 0.85–0.95 and for probable bvFTD between 0.75–0.85 [8,9]. In a small (N = 30) clinical diagnosis based study [10] the sensitivity as high as 1.00 for possible bvFTD and that of 0.60 for probable bvFTD were detected, but it is possible that these values may be overestimates due to selection bias i.e. the inclusion of patients among whom the disease was already clearly evident. A specific *C9ORF72* core phenotype has been postulated with early behavioural symptoms, early psychiatric contacts and a high frequency of neuropsychiatric symptoms [11–13]. However, the revised FTDC in bvFTD patients with the *C9ORF72* expansion has been evaluated in only one small study (n = 10) [14] which reported low sensitivity estimates i.e. 0.60 for possible bvFTD and 0.38 for probable bvFTD.

Since the phenotype of the *C9ORF72* expansion carriers as well as their core clinical symptoms in bvFTD may differ from those of the other bvFTD patients, we assessed the sensitivities of the FTDC possible and probable bvFTD criteria and evaluated the phenotype in the light of these criteria in 36 Finnish bvFTD patients carrying the *C9ORF72* expansion.

## Materials and Methods

### Standard protocol approvals, registrations, and patient consents

The ethics committees of Kuopio University Hospital and Oulu University Hospital approved the research protocol in accordance with the principles of the Declaration of Helsinki. Written informed consent was obtained from all the participants.

### Participants

We identified 43 patients with the *C9ORF72* expansion from the memory outpatient clinics of Kuopio University Hospital and Oulu University Hospital. Individuals with language variant(s) (N = 7) were excluded. Thus, the final cohort consisted of 36 patients presenting clinical features for bvFTD, i.e. 32 patients with bvFTD (N = 32) and four with bvFTD-MND. The age range was 44–76 years at the time of the first symptoms, 44% (N = 16) were males. BvFTD presented before MND in three out of four cases.

### Clinical review

All *C9ORF72* expansion carriers were evaluated by a neurologist specializing in memory diseases (PH, AMR, and VM). The age at the onset of symptoms and data on behavioural symptoms were registered systematically. Clinical and neuropsychiatric criteria were evaluated during the diagnostic procedure. We had only two sib pairs in this study and both of the sib pairs were referred to neurological examination independently without knowing that there were FTLD cases in their family. The clinical data was compared against FTDC criteria [8]. The overall functional decline over the time was evaluated from all the patient records. Data on neuropsychological evaluation was available from 33 patients. Deficits in executive tasks combined with relative sparing of memory and visuospatial functions were accepted as positive neuropsychological findings for bvFTD. Structural brain imaging was conducted in all patients (MRI = 17, CT = 7, both MRI and CT = 12) and analyzed visually by an experienced neuroradiologist. SPECT or PET was done in 17 patients and analysis was based on visual reads. The presence of frontal and/or anterior temporal atrophy in MRI and/or CT or hypoperfusion frontally and/or anterior temporally in PET/SPECT was accepted as a positive neuroimaging result for FTLD. Psychiatric assessment was done according to prevailing clinical practice by using the concurrent diagnostic criteria, the period spanning few decades. The symptoms in psychiatric records were carefully re-checked by the experienced senior psychiatrist (HKH). Results were categorized into mood (affective) disorders and psychotic (schizophrenia, schizotypal and delusional) disorders.

### Genetic analyses

The *C9ORF72* expansion (>40 repeats) was analyzed using the repeat-primed polymerase chain reaction assay [4].

### Statistical analyses

All the statistical analyses were performed with SPSS 19. The survival of the patients was analyzed with the Kaplan-Meier test. Statistical analysis was 2-tailed and p<0.05 was considered statistically significant.

## Results

The demographic features as well as onset/duration of the patients are presented in Table 1.

**Table 1:** Characteristics of the study participants.

	Years		
	Mean (SD)	Range	95%CI
**Age at the onset of the symptoms**	59.3 (6.6)	44–76	57.1–61.3
**Age at the diagnosis**	61.2 (6.5)	46–79	59.1–63.4
**Duration of the symptoms to diagnosis**	2.1 (1.7)	0–7	1.5–2.6
**Age at the death (N = 18)**	67.1 (6.5)	56–81	63.9–70.3
**Duration from the onset to death**			
Total cohort	6.7 (3.9)	1–13	4.8–8.7
Pure bvFTD without MND (N = 14)	7.8 (3.8)	1–13	5.6–10.0
bvFTD with MND (N = 4)	3.0 (0)		

Ages, standard deviations (SD), ranges and 95% confidential intervals of characteristics of the study participants (N = 36).

**Fig 1:**
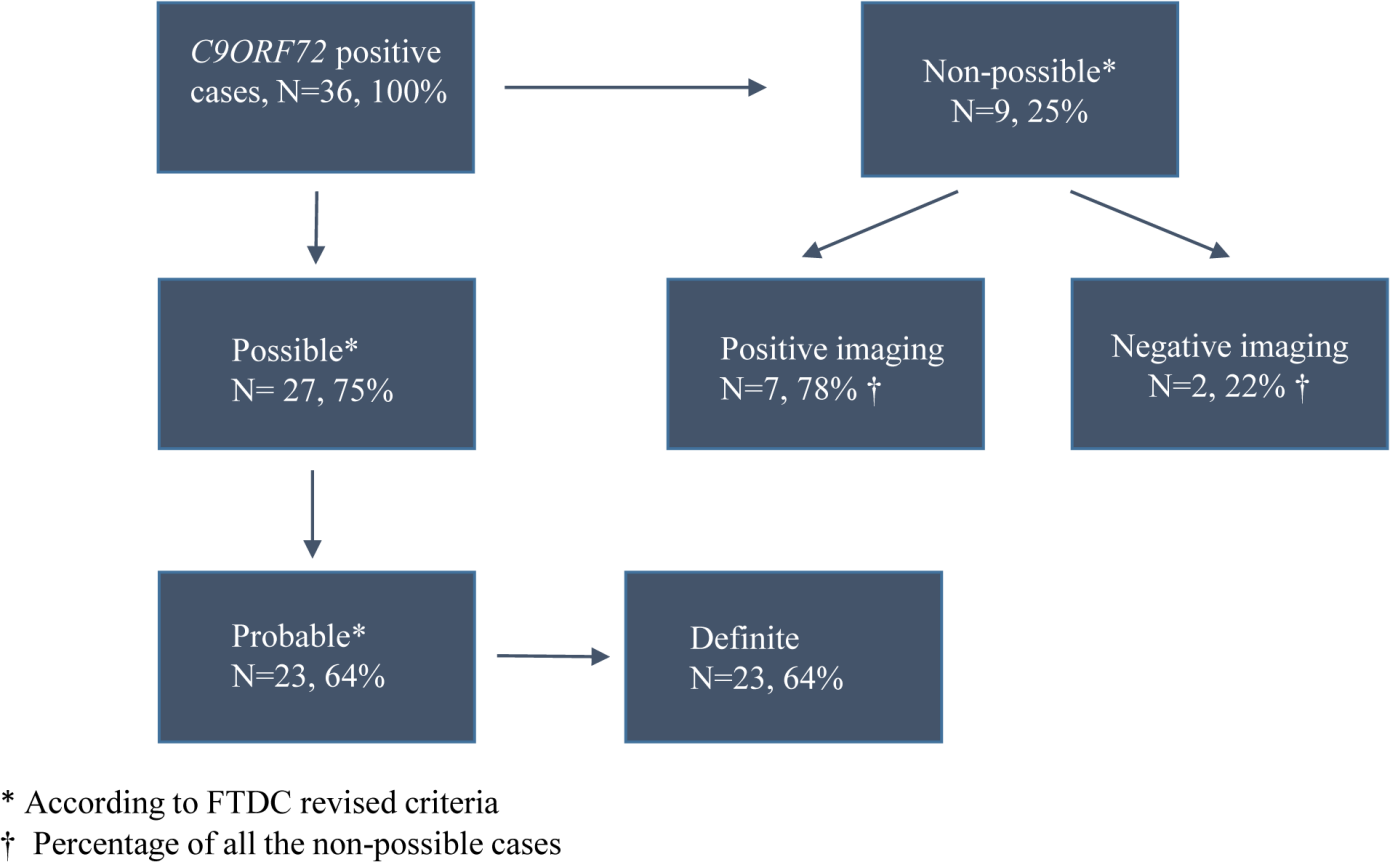
Flow chart of the study. Thirty-six patients with the *C9ORF72* expansion were examined. Seventy-five percent (N = 27) of the study participants met the possible FTDC criteria and 64% (N = 23) met the probable FTDC criteria. Altogether 25% did not meet FTDC possible criteria. Seventy-eight percent of them (N = 7) had positive neuroimaging result and 22% (N = 2) had negative neuroimaging result for FTD.

When the total cohort, i.e. 36 patients with the *C9ORF72* expansion, was assessed 75% of the patients (N = 27) met the FTDC criteria for possible bvFTD (0.75 sensitivity, SD 0.44, 95%CI 0.57–0.87) and 64% (N = 23) met the probable bvFTD criteria (0.64 sensitivity, SD 0.49, 95%CI 0.46–0.79) (Fig 1). If we assessed only pure bvFTD (N = 32), the sensitivity of the possible bvFTD criteria was 0.81 (SD 0.40, 95%CI 0.63–0.92) and sensitivity of the probable bvFTD criteria was 0.69 (SD 0.47, 95%CI 0.50–0.83), whereas these figures for bvFTD-MND (N = 4) were both only 0.25. All of the cases suffered from one or more symptoms of FTLD at the time of diagnosis.

**Fig 2:**
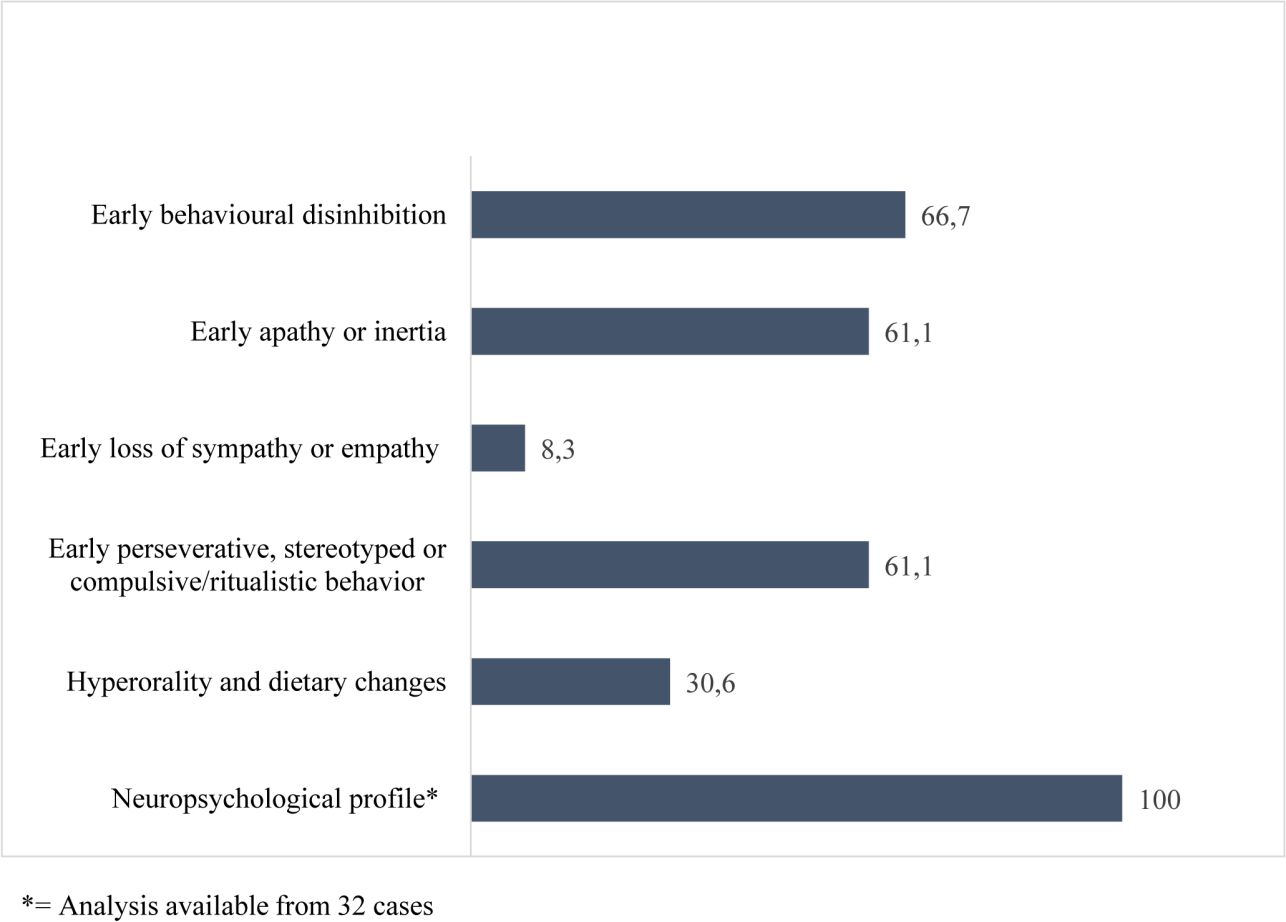
Frequency of behavioural and cognitive symptoms in patients with the *C9ORF72* expansion (%). The numbers at the end of the bars represent percentage frequencies. Neuropsychological examination was done to 32 study subjects out of 36.

**Fig 3:**
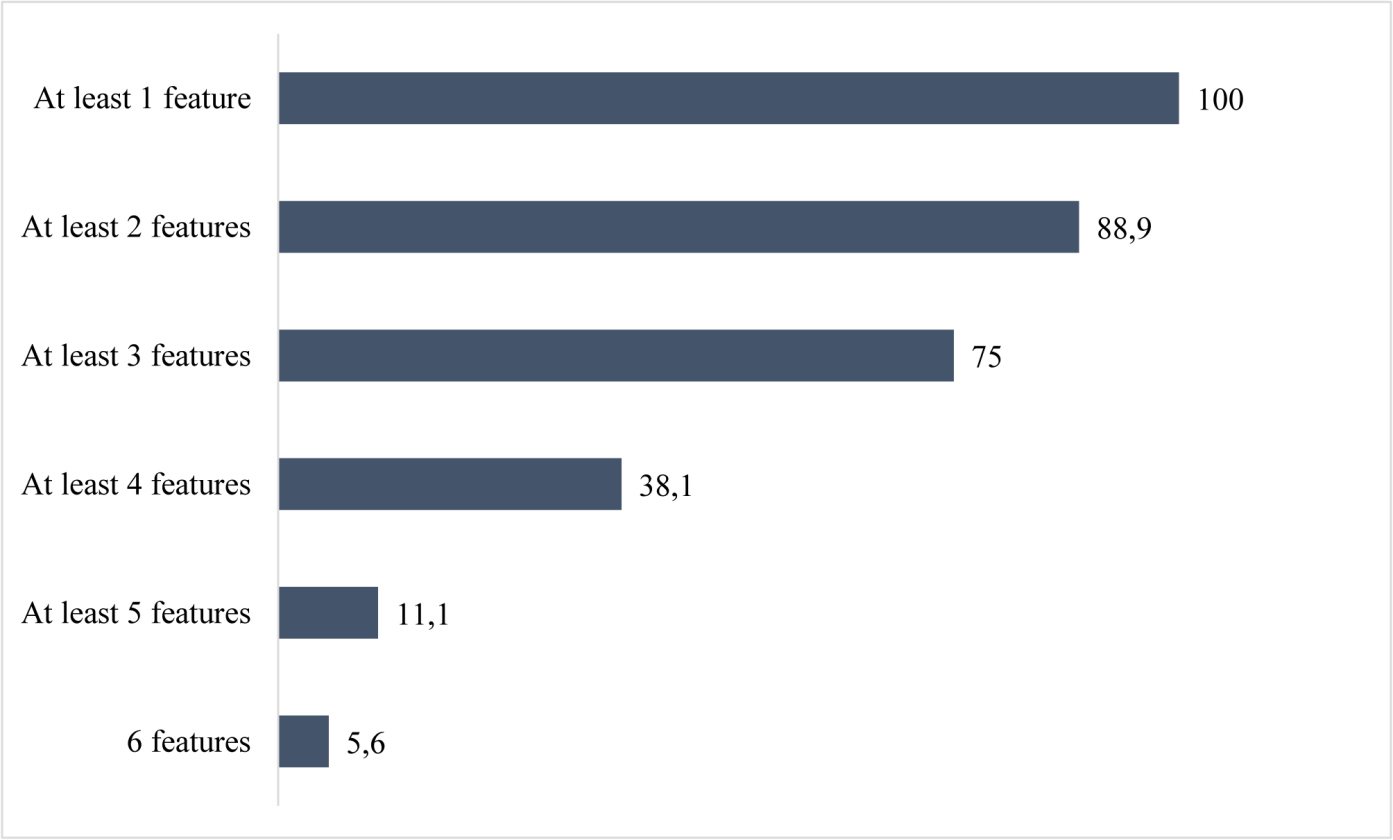
Cumulative percentage of possible features of the FTDC criteria (%) in patients with the *C9ORF72* expansion. The numbers at the end of the bars represent cumulative percentage frequencies of possible bvFTD features. All the study participants had at least one possible feature and 5.6% had all the six features.

The mean number of behavioural and cognitive criteria of possible bvFTD in the total cohort was 3.19 (SD 1.26, 95%CI 2.77–3.62, range 1–6) (Figs 2 and 3). In pure bvFTD patients without MND, the mean number of possible bvFTD features was 3.38 (SD 1.2, 95%CI 2.95–3.80, range 1–6), while in FTD-MND patients, the number of possible bvFTD features was only 1.75 (range 1–3). All bvFTD-MND cases displayed a positive neuropsychological profile for bvFTD, but behavioral changes were absent.

When the neuroimaging and functional decline criteria (FTDC possible bvFTD criteria apart from behavioural features included in the probable bvFTD criteria) were examined, these criteria achieved a slight improvement in the sensitivity for *C9ORF72* expansion carriers i.e. 81% (N = 29) of the patients were found to be positive with these criteria (0.81 sensitivity, SD 0.40, 95%CI 0.63–0.91). However, neuroimaging (PET/SPECT and MRI or CT) alone exhibited the highest sensitivity (0.86 sensitivity, SD 0.35, 95%CI 0.70–0.95). MRI or CT criteria were fulfilled in 26 out of 36 cases (0.72 sensitivity) and PET/SPECT in 14 out of 17 cases (0.82 sensitivity). A diffuse cortical and central atrophy without frontal or temporal predominance was detected in eight cases that did not fulfill the MRI criterion and two remaining cases had normal brain MRI imaging. A bilateral parietal hypoperfusion in PET scanning was detected in one case who did not fulfill PET criterion and PET revealed normal in two of cases.

Psychiatric symptoms were also evaluated in all of the cases. The majority of the patients (61.1%; N = 22) were found to be suffering from these symptoms. Psychotic symptoms without mood symptoms were detected in 30.6% (N = 11) of the patients; 11.1% of the patients (N = 4) displayed only mood symptoms, while 19.4% of the cases (N = 7) experienced both psychotic and mood symptoms. The mean delay from the psychiatric symptoms to diagnosis of bvFTD was 4.6 years (95%CI 0.8–8.4, SD 8.5). In spite of one patient with 40 years of progressive disorder, the delay was less than ten years for the others.

Altogether 25% of the patients (N = 9) did not meet the FTDC criteria for possible bvFTD. This subgroup consisted of three patients with FTD-MND and six with pure bvFTD. Their mean age was at the onset of symptoms 56.6 years (SD 6.4, 95%CI 51.6–61.5, range 44–66) and at the diagnosis 59.3 years (SD 6.7, 95%CI 54.2–64.5, range 46–69), while being 60.2 years (SD 6.5, 95%CI 57.7–62.8, range 46–76) and 61.9 years (SD 6.5, 95%CI 59.3–64.4, range 48–79) among the patients fulfilling the criteria (N = 27), respectively. In addition, the mean number of possible bvFTD features among the patients that didn’t meet the possible bvFTD criteria was 1.56 (SD 0.5, 95%CI 1.15–1.96, range 1–2) and all of them showed symptoms of executive dysfunction in the neuropsychological examination. Apathy or inertia was present in four of them and one patient suffered from disinhibition, but none of the other possible bvFTD criteria were fulfilled by any of them. However, 77.8% of these cases (N = 7) presented with a positive neuroimaging result for FTLD (Fig 1) and 66.7% of these cases (N = 6) were positive for both cognitive decline and neuroimaging. Psychiatric symptoms were present in 55.6% (N = 5) of the cases.

The PET/SPECT was normal in 17.6% of the scanned individuals (three out of 17 patients) and thus it did not meet the PET/SPECT criteria for probable bvFTD. All these cases suffered from pure bvFTD without MND. Their mean age at onset (53.7 years, SD 1.5, 95%CI 49.9–57.5, range 52–55) and at time of death (63.5 years, SD 2.1, 95%CI 44.4.-82.6, range 62–65) were significantly younger compared to all patients (Table 1). Their mean number of possible bvFTD features was 3.3 (SD 1.5 95%CI -0.46–7.13, range 2–5) and in addition, psychiatric symptoms were prevalent in all these cases. On the other hand, structural brain imaging indicated bvFTD in all thesePET/SPECT negative cases. The mean duration of the disease from onset of symptoms to PET/SPECT scanning was 2.0 years among the PET/SPECT negative cases while the mean duration of the disease was 1.6 years among the PET/SPECT positive cases.

The mean age at death in the total cohort was 67.1 years (N = 18, SD 6.5, 95%CI 6.39–70.3, range 56–81).

## Discussion

The clinical diagnosis of bvFTD is rather challenging and the new validated criteria for bvFTD have been recently proposed [8]. However, the sensitivity of these criteria has been evaluated in only a few studies. In the present study, we investigated the accuracy of FTDC criteria in a cohort of bvFTD and bvFTD-MND patients with the *C9ORF72* expansion.

We found that the sensitivity of FTDC criteria among all patients (including bvFTD and bvFTD-MND) was 0.75 for possible bvFTD and 0.64 for probable bvFTD. However, the sensitivity was even higher (0.81 for possible and 0.69 for probable) among the patients with pure bvFTD i.e. excluding those with features of MND. These sensitivities are clearly higher compared to a previously found in a smaller study of ten patients with the *C9ORF72* expansion (i.e. 0.60 for possible bvFTD and 0.38 for probable bvFTD) [14]. Instead, our results more closely resembled those for neuropathologically-confirmed bvFTD cases. In large, neuropathologically-confirmed bvFTD case series assessing FTDC criteria, sensitivities were only slightly higher i.e. 0.85 for possible and 0.75 for probable bvFTD [8] than in the present study. Still, another large autopsy-based study did suggest even higher sensitivities, i.e. 0.95 for possible bvFTD and 0.85 for probable bvFTD [9].

In the present study, the number of the patients with FTD-MND phenotype was limited and the sensitivity of FTDC criteria was very low in these cases (0.25). This low sensitivity in FTD-MND cases may be attributable to the extremely rapid disease progression, short survival of the patients and limited number of cases. The neuropsychiatric symptoms may also be difficult to recognize in patients with bulbar symptoms. Our results are in line with a previous study in which the *C9ORF72* expansion carriers with bvFTD-MND tended to have reduced sensitivity for FTCD in comparison to non-carriers [15].

The mean number of the cognitive or behavioural criteria found in the present cohort was three (3.19). The relatively high probable bvFTD sensitivity in our study may be explained by the high sensitivity of neuroimaging when combined with cognitive and behavioural criteria. Our findings may even indicate that the actual sensitivity of neuroimaging (including MRI or CT combined with PET/SPECT if available) could be even higher (0.86) than the FTDC criteria sensitivity for possible bvFTD (0.75). When examining these values more closely, it is interesting to note that the sensitivity for MRI alone was 0.72 and for PET/SPECT 0.82, which is parallel with a recent study by Snowden and colleagues [16]. This finding is exciting because a previous study claimed that there were very low sensitivities for MRI (0.13) and PET (0.50) among the *C9ORF72* expansion carriers [14]. However, those findings may have been somewhat biased due to small size of the cohort (N = 10). Quite contrary, the sensitivity of SPECT was 0.94 among the *C9ORF72* expansion carriers (N = 16) in another study [16].

The core phenotype in the bvFTD patients with the *C9ORF72* expansion seems to differ from neuropathologically-confirmed bvFTD cases (i.e. without data on their *C9ORF72* status), while the number of diagnostic criteria seems to be lower in patients with the *C9ORF72* expansion. This argument is supported by the finding that in neuropathological and clinical cohorts, the sensitivity of each possible feature is on average very high (over 50%) except for hyperorality [7,9,10,17,18] while Devenney and colleagues found the mean number of possible bvFTD features to be 4.2/6 in patients with the *C9ORF72* expansion which is slightly higher compared to our results (3.19/6 features). Executive dysfunction in the neuropsychological profile was detected in all of the patients and this was found even in the early stages of the disease or in cases that did not fulfill the criteria for bvFTD. Disinhibition, apathy/inertia and early perseveration were also common symptoms, while early loss of sympathy or empathy was rare. The relatively poor sensitivity (0.33) for early loss of sympathy or empathy has also been detected in a previous neuropathologically-confirmed (N = 135) cohort from UK and Australia, [17]. However, when evaluating the absence of early sympathy or empathy, cultural differences may play a role. Thus, the detection of different symptoms may indeed be linked to the cultural environment, e.g. with regard to emotional issues like the loss of empathy or sympathy. This is the first study from Finnish patients and there is no previous data which would have assessed FTDC criteria in either the Nordic or Baltic countries.

Surprisingly, PET/SPECT was normal in a substantial proportion (17.6%) of the patients. Thus, a normal finding in SPECT cannot exclude the possibility that the patient has the *C9ORF72* associated bvFTD. This finding is supported also in previous studies of FTD patients with the *C9ORF72* expansion (N = 10 and 32) [14,16]. Interestingly, the PET/SPECT negative cases in the present study tended to have a considerably earlier onset of symptoms and earlier death, more psychiatric symptoms and more possible features of FTDC criteria, but their number was too low to draw any firm conclusions. None of the PET/SPECT negative individuals suffered from MND. The time of PET/SPECT scanning did not affect the results, because these scans were conducted at clearly later clinical disease stages in the PET/SPECT negative cases than in their PET/SPECT positive counterparts. Furthermore, structural brain MRI imaging had its limitations with 0.72 sensitivity, highlighting the need to combine structural (MRI or CT) and functional (PET/SPECT) neuroimaging, which resulted in a surprisingly high sensitivity (0.86).

To date, this is the largest genetically confirmed cohort assessing the FTDC revised criteria in bvFTD patients with the *C9ORF72* expansion. Patients carrying the expansion seem to fulfill the FTDC possible and probable bvFTD criteria rather well, but the sensitivity was lower as compared to that obtained in neuropathologically-confirmed bvFTD cohorts. Executive dysfunction seems to be a core feature in all of the patients and psychiatric symptoms are abundant, but the number of the behavioral symptoms is fewer than encountered in neuropathologically-confirmed cohorts without genetic confirmation.
